# Biosensors—Publication Trends and Knowledge Domain Visualization

**DOI:** 10.3390/s19112615

**Published:** 2019-06-09

**Authors:** Nasrine Olson, Juhee Bae

**Affiliations:** 1 Swedish School of Library and Information Science (SSLIS), University of Borås, 501 90 Borås, Sweden; 2 School of Informatics, University of Skövde, 541 28 Skövde, Sweden; Juhee.Bae@his.se

**Keywords:** biosensors, bibliometrics, scholarly publications, emerging trends

## Abstract

The number of scholarly publications on the topic of biosensors has increased rapidly; as a result, it is no longer easy to build an informed overview of the developments solely by manual means. Furthermore, with many new research results being continually published, it is useful to form an up-to-date understanding of the recent trends or emergent directions in the field. This paper utilizes bibliometric methods to provide an overview of the developments in the topic based on scholarly publications. The results indicate an increasing interest in the topic of biosensor(s) with newly emerging sub-topics. The US is identified as the country with highest total contribution to this area, but as a collective, EU countries top the list of total contributions. An examination of trends over the years indicates that in recent years, China-based authors have been more productive in this area. If research contribution per capita is considered, Singapore takes the top position, followed by Sweden, Switzerland and Denmark. While the number of publications on biosensors seems to have declined in recent years in the PubMed database, this is not the case in the Web of Science database. However, there remains an indication that the rate of growth in the more recent years is slowing. This paper also presents a comparison of the developments in publications on biosensors with the full set of publications in two of the main journals in the field. In more recent publications, synthetic biology, smartphone, fluorescent biosensor, and point-of-care testing are among the terms that have received more attention. The study also identifies the top authors and journals in the field, and concludes with a summary and suggestions for follow up research.

## 1. Introduction

With advances in biosensor technologies, we have witnessed rapid growth in related research, publications, use domains, and application areas in multiple fields including drug discovery and biomedicine. It is difficult to stay abreast of developments in biosensors or to build an informed, up-to-date overview of related research due to the complexity of the topic and the growing numbers of publications, sources, and research areas. In this paper, we utilize various bibliometric analyses in order to provide an overview of the trends in scholarly publications related to biosensors.

Biosensors are described as “analytical devices that use a biological or biologically derived material immobilized at a physicochemical transducer to measure one or more analytes” [1] (p. 3942). In other words, a biosensor can be described as a device that “utilizes the specificity of a biological molecule to convert a biological signal into an optoelectronic, electrochemical, or piezoelectric signal” [2] (p. 3084). In this way, specific biological processes can be monitored to provide detailed insight into physiology, as well as pathophysiology.

Although it is not easy to identify a definitive starting point for biosensors, early inventions began more than half a century ago. In an early paper [3], an account of instruments that could indicate the chemical composition of blood is given, and blood chemistry of patients has been detected and recorded using an invented electrochemical detection system. When searching for early publications that include the actual term biosensor(s), one can only find a couple of items from 1966 (i.e., [4,5]) and a few more from the 1970s (i.e., [6,7,8]). It is in the 1980s that a larger number of items begin to appear. Since those early days, scholarly publications on biosensors have exploded and today one can find, for example, over 55,000 related items on the PubMed database.

With the growing number of articles that are published on the topic, it is no longer straightforward to build an informed overview of this extended body of publications. In a separate study (not yet published), we have identified over 700 overview articles by experts in the field, which are based on sub-selections of publications. Those overviews provide rich discussions of narrower sub-areas, for example on *graphene-based biosensors* (e.g., [9,10], *carbon nanotube-based biosensors* (e.g., [11,12]), *implantable biosensors* (e.g., [13]), *surface plasmon resonance* (e.g., [14,15]), *chemical biosensors* and application of *electrochemistry for sensing* (e.g., [16,17]) and more. However, while these selective sub-area overviews are useful and informative, with rapidly evolving discoveries and inventions in this field, it also becomes relevant to form an understanding of the trends, patterns of growth, and up-to-date overview of the full set of publications. Bibliometric analyses (that focus on related statistical data to inform of new trends) have been shown to be useful for the latter.

This study came about due to an expressed need for an overview of the developments in biosensors-related publications based on consistent terms that would allow logical comparisons of the topical growth over the years to be made. With that as a starting point, this paper aims to identify and map potential patterns in the production of scholarly publications on biosensors and to offer insights into the subdomains of biosensors research. Accordingly, bibliometric methods are used to investigate the following research questions:

1. How has the number of publications on the topic of biosensors evolved over time? (Section 3.1)
2. Which authors and countries are most active in producing publications on the topic and in which journals are most publications found? (Section 3.3, Section 3.4 and Section 3.5)
3. What are the core keywords in biosensor publications? (Section 4)
4. Are there any identifiable sub-domains in the research related to biosensors, and if so, which? (Section 4.1, Section 4.2, Section 4.3, Section 4.4 and Section 4.5)
5. What can be said about the trends in biosensor research? (Section 3, Section 4 and Section 5)

Measuring objects and events around us is said to be a scientific necessity and a means to making sense of “the complexity of natural phenomena” [18]. Bibliometrics provide us with means to measure science and technology and their outcomes. Over time, various definitions of the term bibliometrics have been put forward by scholars (e.g., see [19]). In general, the term refers to quantitative studies or statistical analysis of scholarly publication data or scholarly publication patterns. Scholarly publications are accompanied by multitude of quantifiable elements that can be used as a basis for analysis. The work by Garfield in creating the Science Citation Index (e.g., [20]) is often recognized as a major influence in the development of the field enabling empirical studies of citations, productivity, networks, impact, and other indicators. Bibliometric methods are often used to provide insights into the cognitive structure of different research fields. There is an abundance of works on bibliometric methods, extending and improving them or discussing potential limitations. There are also extensive publications that apply such methods in studies of formal scholarly communications in different fields and research areas. However, bibliometric studies of publications on biosensors remain very limited.

Through an extensive search for earlier, related bibliometric studies, only half a dozen articles were identified, and none had the same focus or was based on a similarly full range of data as this study. For example, with an interest in the use of biosensors in medical diagnostics, Sheikh and Sheikh [2] use bibliometrics and patent analysis in order to forecast the potential growth of biosensors for use in point of care diagnostics and Internet of Things applications. The focus is on testing different technology forecasting methods. Trends in patents are depicted, however, an overview of the trends in scholarly publications is not included.

In another paper [21], the trends in global environmental monitoring are studied and the most productive countries in that field (by first authors) are identified (with top being US, UK, Italy, China, Germany). Although biosensors are included due to their role in environmental monitoring, they are not the focus of that study. Similarly, biosensors appear in another study [22], only as a part of publications on techniques for monitoring water quality and detection of microorganisms and metagenomics. Again, in that study the most productive countries in the field ae identified (e.g., China, 36.7%; US, 21.86%; followed by others each with significantly fewer publications). Two others study a way of identifying ‘reverse salients’ [23] and nanobiotechnology as an emerging research domain from nanotechnology [24]. Again, while insightful, the overview structure of publications on biosensors is not the focus.

We did find one paper with the focus on biosensor-related publications [25]; however, the scope of the study was limited to publications in Italy and/or by Italian researchers. In summary, while there are bibliometric studies on a few related topics of interest, we did not find any extended mapping of scholarly publications specifically on biosensors. To bridge that gap, this paper contributes with an extensive bibliometric study of scholarly publications on this topic. We argue that the overview and visualizations offered in this paper contribute useful information for scholars who are interested in progress of the area of biosensors.

## 2. Methodology

De Bellis [26] relates back the origins of quantitative measures, on which bibliometrics are based, to the positivistic sociology of Auguste Comte, William Ogburn, and Herbert Spencer. The purpose of this paper is not to reduce the scientific endeavors and communications to just a series of quantitative measures. Statistical measures of scientific communications, despite their merits, can be flawed, limited, and open to manipulations. However, many earlier attempts of applying bibliometric methods to the studies of publications have proved to be instructive and useful in describing communication and production of knowledge. The basic assumption at the root of bibliometric studies is that scientific publications provide an indication of subject matter of science. In the practice of scholarly publication, one typically builds on earlier knowledge produced by others. Furthermore, scholarly publications often include a number of keywords that define the essential areas addressed. Referral to earlier publications, use of common keywords, or shared conferences and more, create links between published works. Examination of such networks, beyond just the nodes and links as intended here (c.f. [27,28]) can provide insights about the knowledge and works that are being produced.

### 2.1. Distribution Laws

There are two central laws linked to bibliometric methods. Bradford’s law of scattering, or Bradford distribution (e.g., [29,30]), proposes that the journals of a field can be divided into three groups, each containing an equal number of publications, with the first group comprising of very small number of prolific core journals hosting many of the field’s publications. That is, he proposes that there is a kind of power-law distribution related to the journals that publish articles related to a field. Similarly, Lotka’s law on frequency distribution of scientific productivity [31], proposes that in any given field there is a small number of prolific authors that are most productive. Lotka proposed that “the number [of persons] making n contributions is about 1/n^2^ of those making one [contribution]” [31] (p. 323). This law was further extended ([32], see also [33]) and is referred to as the Law of Square Roots where it is said that half of the publications in an area are written by a small number of authors equal to the square root of the number of all the authors who publish in that area.

In other words, these laws state that by identifying the core authors or by accessing the material in the core journals, one should be able to form a reasonable sense of the field. Bibliometric methods offer quantitative measures to identify such indicators.

### 2.2. Selection of Database and the Data Set

To form an understanding of the topical discussions in a field, a study of keywords can be useful. The starting point for this study was to form an overview of the developments in publications on biosensors based on uniform and consistent keywords to provide a level of stability in the comparisons over time. While author keywords are very useful and insightful, the choice of terms by individuals can vary from paper to paper and author to author. For example, different authors can use different keywords to indicate *biosensors* as a main topic in their papers. Therefore, an early decision in the study became to analyze trends based on a uniform set of keywords. That, in combination with other criteria (e.g., the extent and availability of relevant data, ease of access for multiple downloads and experimentation, etc.), defined the choice of database to become PubMed due to the presence of its MeSH terms.

PubMed is a free resource offered by the National Centre for Biotechnology Information (NCBI). It comprises over 28 million bibliographic records for biomedical literature from MEDLINE, and other life science journals, as well as online books (https://www.ncbi.nlm.nih.gov/pubmed/). PubMed allows easy downloading of large sets of bibliographic records in one go, a function that is often restricted in other databases. More importantly, the PubMed database provides a uniform indexing of biomedical literature. The Medical Subject Headings, or MeSH terms, form a controlled vocabulary or a specific set of terms that describe the topic of a paper in a consistent and uniform fashion. In PubMed, even author keywords are also available (since 2013–in the field *Other Terms*). The main advantages of using PubMed for this study were the presence of uniform MeSH terms and ease of access to, and retrieval of large sets of data. The disadvantages of using PubMed include: (a) the lack of citation information, (b) a somewhat skewed topical presence, where biomedical applied biosensors publications are privileged; hence, as a result the number of items included in this database are limited as compared with other databases such as Web of Science or Scopus and (c) at times, a lesser complete metadata for some items (e.g., lack of affiliation information for all authors prior to 2014) as compared with the information available in other databases.

With that said, the dataset that forms the empirical basis for this paper comprises of bibliometric data related to 55,971 publications on biosensors. The data set was retrieved from PubMed on 2 March 2018, based on the following search terms:

(((biosensor) OR biosensors) OR bio-sensor) OR bio-sensors

(It should be noted that the internal processes of search conducted on PubMed is somewhat different to others. In PubMed, wild-cards for concatenation or Boolean operands are not typically used).

This search string in turn was expanded by PubMed to become:

(((“biosensing techniques”[MeSH Terms] OR (“biosensing”[All Fields] AND “techniques”[All Fields]) OR “biosensing techniques”[All Fields] OR “biosensor”[All Fields]) OR (“biosensing techniques”[MeSH Terms] OR (“biosensing”[All Fields] AND “techniques”[All Fields]) OR “biosensing techniques”[All Fields] OR “biosensors”[All Fields])) OR bio-sensor[All Fields]) OR bio-sensors[All Fields]

However, considering the issues mentioned above, additional information was gathered to allow for the inclusion of some missing data and comparison between the findings from this dataset and other sources. The additional data was captured on three different occasions from the database Web of Science (WoS), Core Collection, with these specification in all three instances: *Timespan: All years. Indexes: SCI-EXPANDED, SSCI, A&HCI, CPCI-S, CPCI-SSH, ESCI*. The three instances of retrieval were as follows: 2 March 2018 (to see if there is support for the declining numbers even in WoS), 18 January 2019 (to complement with additional country data where this information was missing in the PubMed dataset), and 8 May 2019 (for most recent updates of the publication trends up to the date of submission). The searches conducted in January 2019 involved cross referencing those items in the PubMed dataset that lacked affiliation or country data, with items in WoS using available fields such as title, author, doi and more and even by manual means. With these efforts, the number of items with no author country information was reduced from 1989 to 650. The searches in WoS, in May 2019 were multiple as listed in Table 1.

### 2.3. Bibliometric Methods Used

Typical bibliometric analyses include publication counts, co-citation analysis, co-word analysis and so on (e.g., [34]). Lack of citation data in our dataset eliminates various citation analysis, however, multiple other bibliometric analyses are conducted as presented here.

*Publication counts* indicate “the volume of scientific and technical output” and provide a descriptive measure of levels of activity and productivity in a given field [18] (pp. 158–159), hence can be considered as a quantification of peer review process [34]. In this study, numbers are identified for publication year, types, countries, authors, and more. Together, these results provide an overview of publication patterns over time (similar to other studies e.g., [35,36]) providing indications about where related research is conducted, by whom and to what level.

*Co-word analysis* involves co-occurrences of words in publications, whether in keywords assigned by authors (uncontrolled) and by professional indexers (controlled), or words in titles, abstract or full texts. These word co-occurrences, as stated by Tijssen and Van Raan “reflect the network of conceptual relations from the viewpoint of scientists and engineers active in the field”. By placing the words in context and in relation to other words and concepts, co-word maps can be viewed as “semantic representation of knowledge structures” [37] (p. 105).

*Classification analysis* refers to co-occurrences of terms used in bibliographic databases to group together publications for easy access and retrieval. Such information items could provide further information about a document’s topic or specialization area or the field. An example could be subject classification terms, which have “a well-defined and consistent meaning over the entire knowledge domain; this makes them particularly attractive for studying and depicting the main cognitive structure across large scientific and technological area” [37] (p. 105).

### 2.4. Tools and Pre-Processing

There are numerous tools available for bibliometric studies. We chose to use VOSviewer (by Nees Jan van Eck & Ludo Waltman; e.g., see [38]), a freely available user-friendly tool, commonly used for constructing and visualizing bibliometric networks. It enables typical bibliometric analyses such as co-authorship, co-occurrence, and more.

Notably, there are often inconsistencies in data that need to be resolved before any analysis. For example, a country’s name can be written slightly differently (e.g. UK, United Kingdom) and hence be treated as two different countries by the tools used. Some pre-processing was conducted in Excel. For the analysis in VOSviewer, some clean-up of data was done by the use of the thesaurus option offered within the VOSviewer where one can instruct the tool to treat, for example, “biosensor” and “biosensors” as the same term so that both terms are processed as the collective of “biosensor(s)”.

We used geotext (https://github.com/elyase/geotext) to separate the first author countries from the affiliation information which included all the information of multiple authors. Geotext extracts country and city mentioning from text information. However, country information was lacking for 1989 authors, we therefore found and added some of the missing information (i.e., in 1339 items) by conducting searches in WoS and with some manual work.

Furthermore, we used a utility created at our request (which is now included in the ScienceScape collection http://tools.medialab.sciences-po.fr/sciencescape/) called *Medline/PubMed to CSV*.

## 3. Trends Analysis Results

A study of publications and publication patterns can provide insights into the state-of-the-art research conducted within a field. We have conducted multiple analyses related to patterns of publications over the years, including publication types, countries, journals, authors and keywords. In this section we present our findings under related sub-headings.

As indicated in the analyses that follow immediately after this short introduction, there is an indication that there exists a time lapse in inclusion of the more recent research output in the PubMed database. This is not, however, the case if data from other databases such as WoS are considered. Therefore, although all the analyses of the PubMed data are based on the full dataset, the related figures only depict the results up to 2015 to avoid confusion about the sharp dips thereafter which are potentially based on the delays in registration.

### 3.1. Trends in Scholarly Publications on Biosensors—General Overview

**Figure 1:**
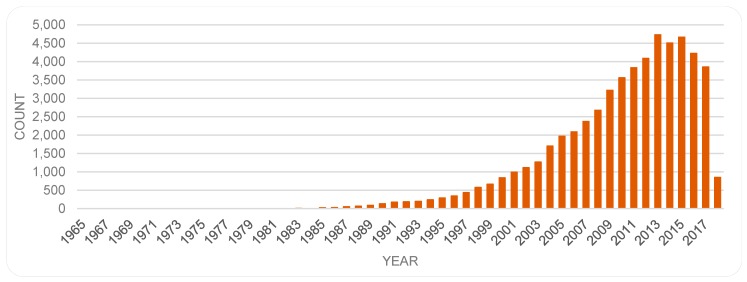
Yearly number of publications on biosensors based on the PubMed dataset.

The number of publications on a topic represents the volume of scientific output. As discussed by other researchers, scientific publishing is growing in general and at slightly varying rates in different fields (e.g., [39,40]). When it comes to publications on biosensors, the number has increased over the years, from just a handful over the first two decades, to well over 3000 items per year since 2009 (c.f. [2] regarding the number of patents; or c.f. publication trends in [22]). As mentioned, the main data for this study was retrieved in March 2018 from the PubMed database. That dataset suggests a decline in the number of publications on biosensors in the more recent years, with a peak in 2013 with 4729 items, as shown in Figure 1.

However, it should be noted that there is typically a time lapse in registration and inclusion of recent publications in databases. Furthermore, the speed of registration may differ from database to database. To examine this decline in numbers further, additional data was captured at the same time (March 2018) from the WoS database for the sake of comparison. The data from WoS did not support the above findings and recent decline, but rather indicated continued growth even in the more recent years. There may be several explanations for this discrepancy. There may be a slower rate of inclusion of new items in the PubMed database as compared to WoS. An alternative explanation could be that although the total number of publications on biosensor(s) is on the rise, the number of publications that correspond to the interest areas of PubMed may be declining. This issue needs further investigation.

**Figure 2:**
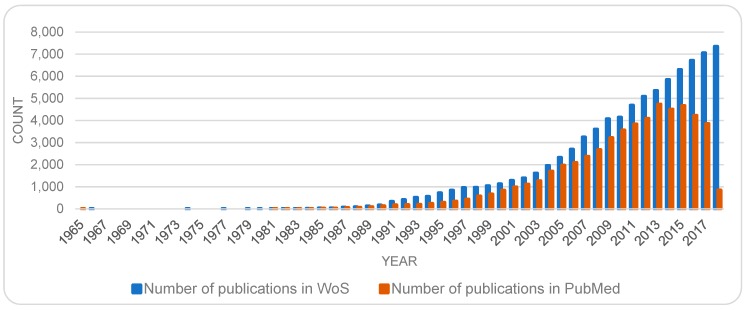
Yearly publication counts on biosensor(s) based on Web of Science data captured in May 2019. The publication trend based on the original PubMed data from 2018 is also included as a point of reference.

Meanwhile, although this study is mainly based on data retrieved from the PubMed database in 2018, for an up-to-date overview of the trends, additional new data (8 May 2019) was captured from the WoS database. Figure 2 depicts the trends based on this new set of data. (The trend from the original PubMed data is also included as a point of reference.)

**Figure 3:**
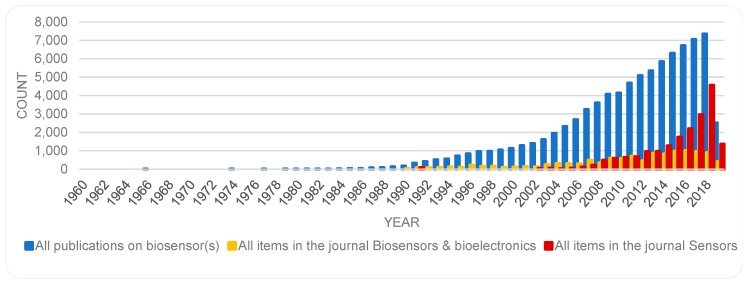
Yearly number of publications on biosensors (85,174 items) as compared with total publications in the journals *Biosensors & bioelectronics* (11,794 items) and *Sensors* (18,997 items) based on data retrieved from WoS on 8 May 2019.

It may also be of interest to see how the publications on biosensors compare, in relative terms, with other trends. For this, further data (8 May 2019) was captured from WoS on publications on biosensors and all the publications on any subject in the two journals of *Biosensors & bioelectronics* and *Sensors*. The findings are collated in Figure 3. As shown, the number of publications on biosensors (85,174 items) is larger than total number of publications in either of the journals *Biosensors & bioelectronics* (11,794 items) or *Sensors* (18,997 items). The publications on biosensors (i.e., the numbers in total regardless of the source of publication) have increased on an even pace. As shown, the growth rate of publications in the journal *Sensors* follows a steeper slope while the number of publications in the journal *Biosensors and bioelectronics* has fluctuated over the years.

**Figure 4:**
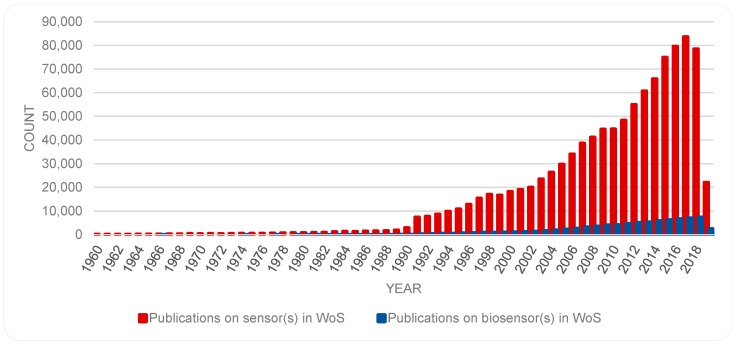
A comparison between the publications on biosensors as compared with publications on sensors.

Another topic with close affinity with biosensor(s) is that of sensor(s). Further new data (from WoS on 8 May 2019) was captured regarding the number of publications on the topic of sensor(s) which resulted to 1,039,670 items. The growth of the number of publications on both topics is illustrated in Figure 4.

**Figure 5:**
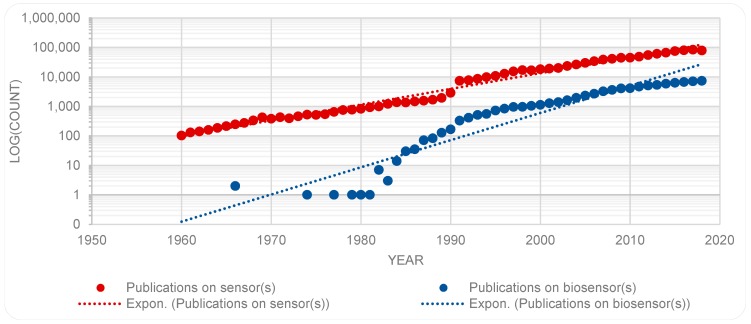
A comparison between the publications on biosensors as compared with publications on sensors (semi logarithmic scale).

While publications on both topics are on the rise, there are some fluctuation, most noticeably in the number of publications on the topic of sensor(s). To get a sense of the growth in an area, some scholars have examined the trends of cumulative number of publications (c.f. [40]), however, others (e.g., [39]) have found an analysis of numbers per year more instructive. Accordingly, Figure 5 provides a semi logarithmic presentation of the number of publications, for each year, for each of the topics sensor(s) and biosensor(s). Here, the fluctuations in the publications on biosensors become more noticeable, and in both cases a surge in numbers of publications is marked in the early 1990s; more so and for a longer period in the case of biosensor(s). While Figure 2 and Figure 3 show a continuous rise in the number of publications on biosensors, Figure 5, suggests a showing down in the growth in the recent years. This trend should be re-examined at a later date to remedy the time lapse involved in registration of new items in databases.

When it comes to analyses of publication numbers as a measure of scientific output, a few issues should be observed. For one thing, the research output cannot solely be measured by the number of items found in traditional databases. Presence of open access repositories, online publication practices, and more, have changed the traditional publication methods and have added to the complexity of the matter. Furthermore, number counts assume an equal value for all publications, which is not always the case. Regardless, analyses similar to what is presented above are still seen as valuable and informative and do provide some indications about the research output trends in specified areas.

### 3.2. Publication and Support Types

The field ‘publication type’, contains 54 unique values; some of the terms are those typically used to describe the forms of publications (e.g., *Book Chapter, Journal Article*, etc.), others are terms that are more descriptive of the contents, (e.g., *Case Report*; *Observational Study*) or research support types, (e.g., *Research Support, U.S. Gov’t, P.H.S.*, etc.). Many of the items are given multiple publication types, although the orders vary from item to item (e.g., “*Journal Article*|*Review*”, for some and “*Review* | *Journal Article*” for others).

**Figure 6:**
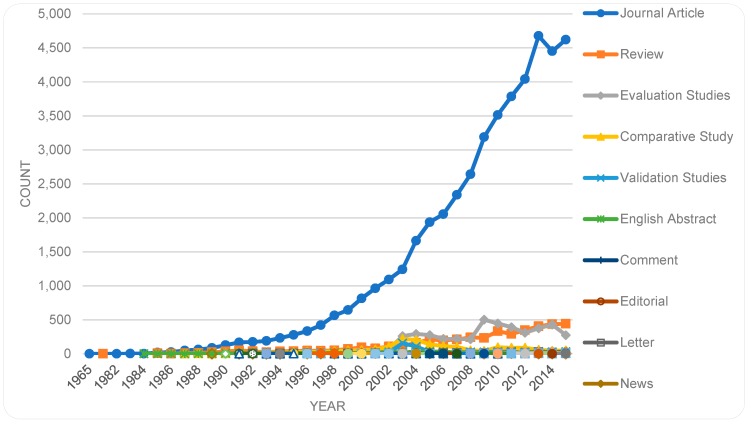
Publication types and their trends over the years.

We separated any types of research support (43,221), from our main findings and examined separately. Since a publication can be assigned multiple types, all publication types besides research support were identified and their trends illustrated in Figure 6. As shown, *Journal Article* is the publication type most occurring (46,414), whether in the first or in alternative positions. In the remaining 12,849 items, the top five publication types are *Review* (4382 items), *Evaluation studies* (4311 items), *Comparative study* (1771 items), *Validation studies* (532 items), and *English abstract* (523 items), with the remaining 40 types each occurring in smaller numbers.

**Figure 7:**
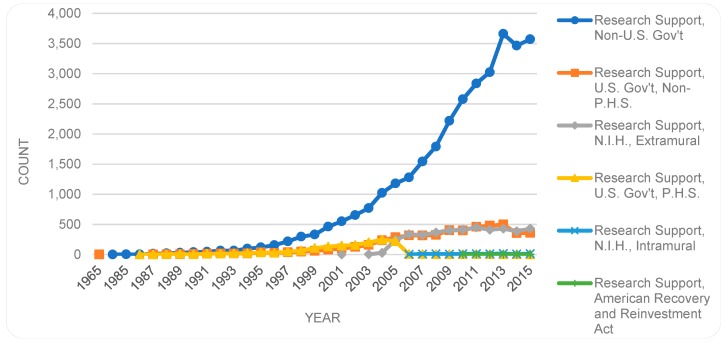
Support types and their trends over the years.

Figure 7 illustrates the research support types, which are included as acknowledgment of a support (e.g., funding) from a particular institute. As indicated, research support is mostly from ‘non-U.S. government’ sources. As shown, in Figure 6 and Figure 7, a somewhat similar developmental curvature exists for the top publication type (*Journal Article*) and the top research support type (*Research Support, Non-U.S. Gov’t*).

### 3.3. Languages and Countries

As can be expected of this dataset, publications in English dominate by far (98.7%), followed by Chinese (0.41%), Russian (0.38%), and Japanese (0.23%). The numbers of publications in other languages were minimal.

For a study of the countries, one can extract country information from the field *place of publication.* This provides information about the countries in which different papers are published. If one is interested in the countries in which the research has been conducted, the situation becomes more complex. Should one examine the nationality of the authors, the country of the funding organization, or the country of author affiliation? It is most common to go by the latter. However, some complications remain since at times an author may have multiple affiliations, (raising the question about which country to include) or in some cases, the affiliation information is fully or partially missing, for one or more of the authors. Furthermore, when a paper is co-authored some choose to look at the first author, while others choose to use a fractionalized counting where the credit is subdivided equally among all the countries involved. That is, for a paper that has five authors from five different countries, the country of each author would be assigned a value of 1/5th. Even this is problematic, since this treatment assumes equal contribution by all authors which is not always the case. We chose to examine the countries in two ways: the country information extracted from (a) the fields *PL—Place of Publication* (i.e., the country of the source), and (b) *AD—Affiliation* (i.e., the countries of authors), however, only the country of *the first author*. The reason behind this choice was two folded. The first authors typically act as the driver of the publication. More importantly, we made this choice in order to be consistent in the application of the chosen method for the whole period included in the dataset. In PubMed, only the affiliation of the first author is included prior to 2014 (Please see: https://www.ncbi.nlm.nih.gov/books/NBK3827/: “*Multiple affiliations were added to citations starting from 2014, previously only the first author’s affiliation was included*.”).

Based on the *Place of Publication* data, we find that the US and the UK are the dominant countries, indicating that most of the related journals and publication sources (in this dataset) are located in these two countries.

**Figure 8:**
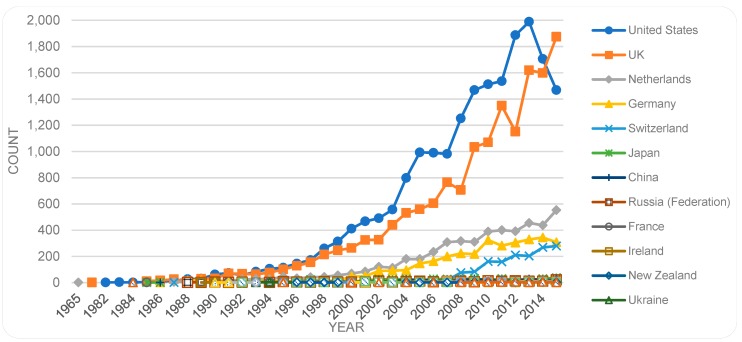
Place of publication (country of the source) by year.

Figure 8 shows the trends, where publications in most of the related countries increase over the years except for the US with decreasing numbers since 2014.

**Figure 9:**
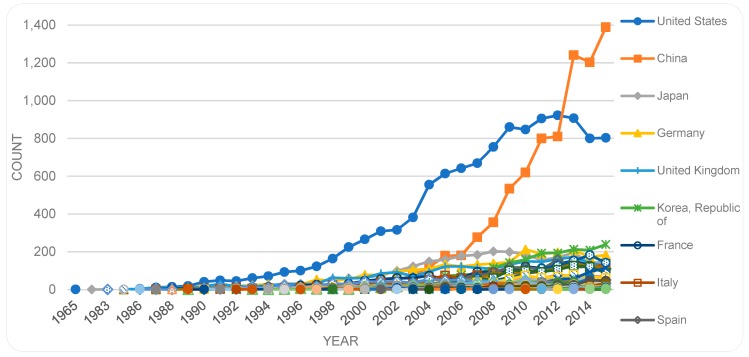
Countries in descending order of publication counts with more than total number of 500 by year (based on countries of the first authors).

We also analysed the countries extracted from the affiliation information of the first authors. While the *publication sources* are situated mainly in the US and the UK, this analysis indicates a strong presence of authors from other countries. Researchers located in China, especially, become noticeable in the second position in total (see Table 2), taking the first position in the more recent years (Figure 9). Table 2 lists the countries (of the first authors) with more than 500 publications (total sum over the years) each, in descending order. Figure 9 shows the trend of the country of the first author over the years. As depicted, the presence of first authors situated in China has increased rapidly while the presence of first authors in the US has been declining in the recent years. This indicates a rather early overtake of publications by Chinese authors, which is in line with reports that China in general has increased its scientific publication and that it is rapidly becoming the largest contributor to global science (e.g., [41]).

Comparing countries of publication sources with countries of first authors, we find that despite the placement of a large number of sources in the UK and the Netherlands (Figure 8), the contributions by authors from those countries (Table 2) are less prominent, i.e., those countries host a larger portion of the publishing industry but contribute with a lesser portion of publications.

**Figure 10:**
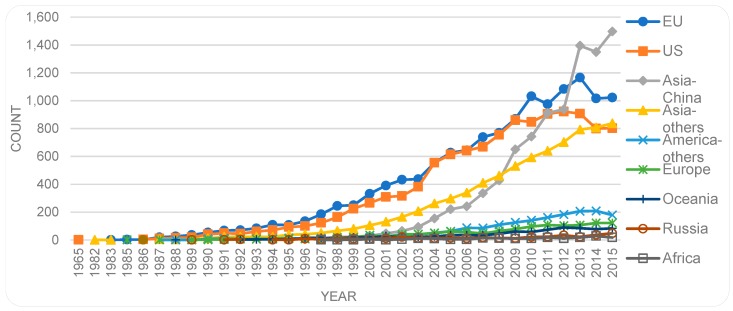
Regional publication trends. Counts of countries of the first authors, grouped in representative regions by year.

Considering the size of population in different countries, it would be informative to also compare research output by the collective of the EU countries with those of the US and China. To get a sense of this, we grouped the data on the location of the first authors (e.g., European Union or other representative regions). By this grouping, we find that the EU becomes dominant with the majority of publications (in total over time), followed by the US, China, and Asian countries other than China. America-others include Canada, central, and southern American countries and Oceania include Australia and New Zealand. Based on this dataset, the publications by both the EU- and US-based first authors have slowed down from 2014 (Figure 10), but publications by first authors from other Asian countries have gradually increased.

**Figure 11:**
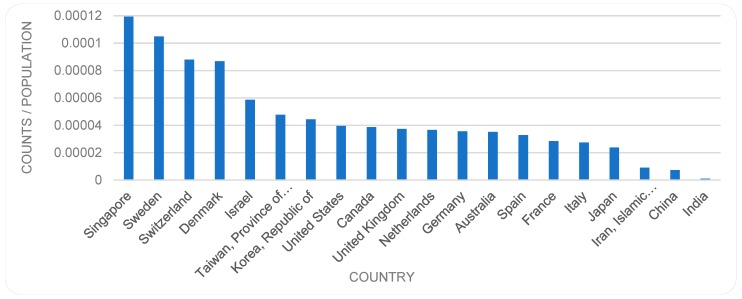
Publication trends per capita.

Indeed, consideration of the size of countries or regions and associated population provides further insights. That is, if we divide the number of contributions by the size of population in each country, the level of contributions by each country becomes rather different to the order presented in Figure 9. The publication numbers per capita are presented in Figure 11, placing Singapore as the most productive country (per capita) followed by Sweden, Switzerland and Denmark. The US that topped the order in Figure 9 is now ranked number 8 and China is at position 19. Japan that had appeared in position 3 directly after China, now take the position 17 ahead of China. Even Iran that was not included in Figure 9, is now included ahead of China.

### 3.4. Journal Titles

**Figure 12:**
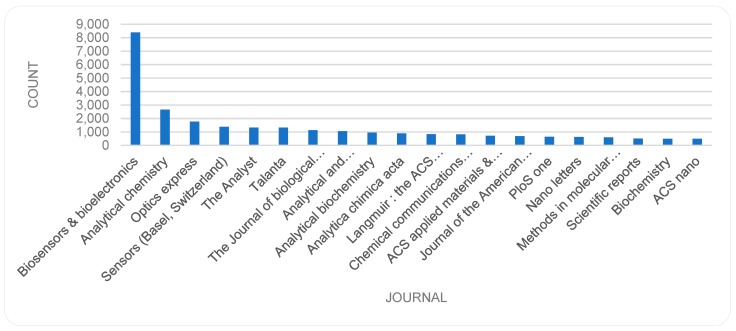
Journal Titles (top 20).

**Figure 13:**
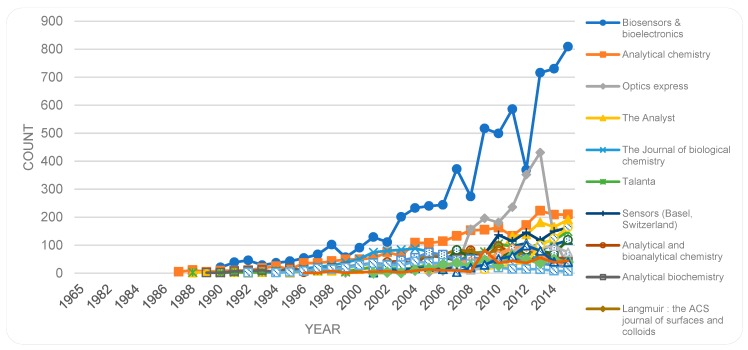
Journal Titles (top 20) by year.

As mentioned in Section 3.2, most of the items in the dataset are of type *journal article* and hence published in a journal. The journal of *Biosensors & bioelectronics* (ISSN: 0956-5663|1873-4235 (Electronic)) is the dominant source of publications followed by *Analytical chemistry, Optics express* and *Sensors*. Figure 12 shows the top 20 journal titles in descending order (a reminder of Bradford distribution) and Figure 13 shows the trend of these top 20 sources. The number of publications in *Biosensors & bioelectronics*, *Analytical chemistry*, *The Analysts*, and *Sensors* has been increasing steadily, while *Optics express* shows a peak and then falls in year 2013. The strong fluctuations for some journals (e.g., Biosensors and bioelectronics) are somewhat puzzling (Figure 13) and would require further investigation in future studies.

### 3.5. Key Authors

**Figure 14:**
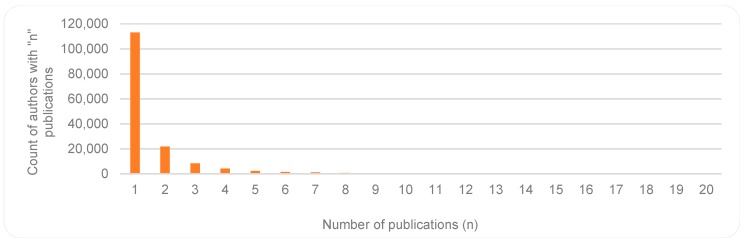
Many authors with few contributions—Few contribute to only one or a few publications on the topic.

**Figure 15:**
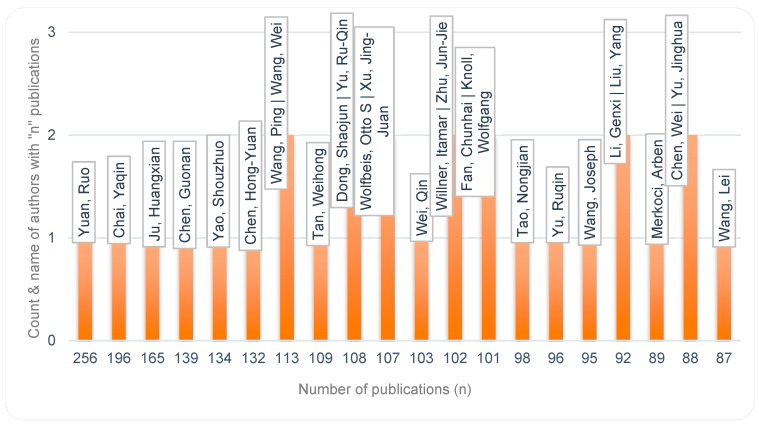
Smaller number of authors with large number of publications - Few authors contribute with many publications on the topic.

Aligned with Lotka’s law, we found an inverse correlation between authors and number of contributions by each author. At one end of the scale, many authors (112,974) have contributed with only one publication; at the other end of the scale a few authors (For author analyses, care was taken for accuracy, e.g., by using full author names and attention to author id numbers where available. However, not all publications included full names or author identification numbers, and since affiliations may vary over times, they were not always useful in identifying all variations in author names) (18) have contributed to more than 100 publications each. Figure 14 and Figure 15 present the two ends of the spectrum of this reverse relationship. With an average of 5.2 authors per publication, one item has 151 authors [43], and there are 1924 items with a single author.

In some disciplines, the main author is listed first, with the rest of authors appearing in descending order based on the level of their contributions. In other disciplines, the last position in the list of authors is just as important as the first position, and is typically assigned to the most senior researcher in the group. Regardless of discipline or level of seniority, the first authors typically play a central, or driving role in the production of the articles. To get a sense of the most frequently occurring authors in this dataset, we examined several core papers by Yuan, Ruo (e.g., [44,45,46]) the top author in the dataset. With 256 publications, Yuan, Ruo appears as the first author in two publications (i.e., [47,48]) and as the last author in a further 92 items. In second place, with 196 publications Chai, Yaqin (e.g., [45,49]), does not appear as the first author at all, but is listed as the last author in 32 items. Those authors in the list with a larger number of publications who also appear as the first author are Wang, Wei (1st author in 30 papers), Liu, Yang (1st author in 26 papers), Wang, Joseph (1st author in 21 papers; also appears as the last author in further 51 items), Wolfbeis, Otto S. (in 14 papers), and Chen, Wei (in 12 papers).

**Figure 16:**
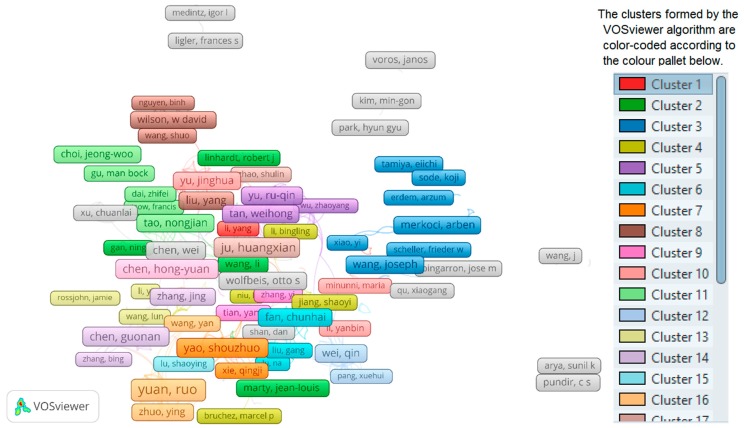
Co-authorship based on authors. The clusters formed by the VOSviewer algorithm are colour-coded according to the colour pallet shown on the right-hand side.

To get a sense of collaborations between different authors, a co-authorship analysis was conducted using VOSviewer. VOSviewer tool creates maps and visualization of network data based on sophisticated clustering and analysis algorithms [38,50,51]. Within the tool, the minimum number of documents per author was set to 5 and documents with more than 25 authors were excluded. Out of 155,446 authors, 8923 met the threshold and we selected the top 500 with the highest co-authorship links. The co-authorship relations form 27 different clusters as calculated and visualized by the tool; this is shown in Figure 16. The top clusters are differentiated using the colors as indicated in the small color pallet on the right-hand side. These clusters include from 2 (cluster 27) to 35 (cluster 6) authors each. The top authors in the data set, Yuan, Ruo, is found in cluster 17, with 12 other authors. To exemplify, and show the list of some the main collaborators, Table 3 lists the top ten authors (based on “the total strength of the links of an item with other items” as further elaborated in VOSviewer manual: http://www.vosviewer.com/documentation/Manual_VOSviewer_1.6.11.pdf) for the first five clusters.

## 4. Keyword Analysis Results

PubMed includes two types of keywords, MeSH Terms and Other Terms (which consist mainly of author keywords from 2013 onwards). The advantage of MeSH terms is their uniformity; however, the author-defined terms provide a level of flexibility and make possible the introduction of terms that have not yet been included in MeSH terms. This paper includes an examination of both MeSH and Other Terms.

### 4.1. MeSH Terms—Occurrence Trends

**Figure 17:**
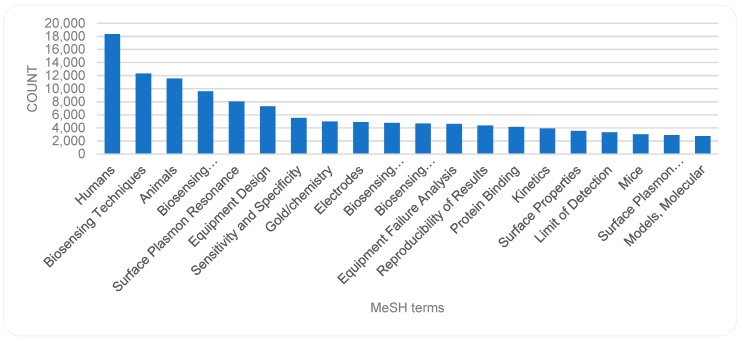
MeSH terms (top 20).

**Figure 18:**
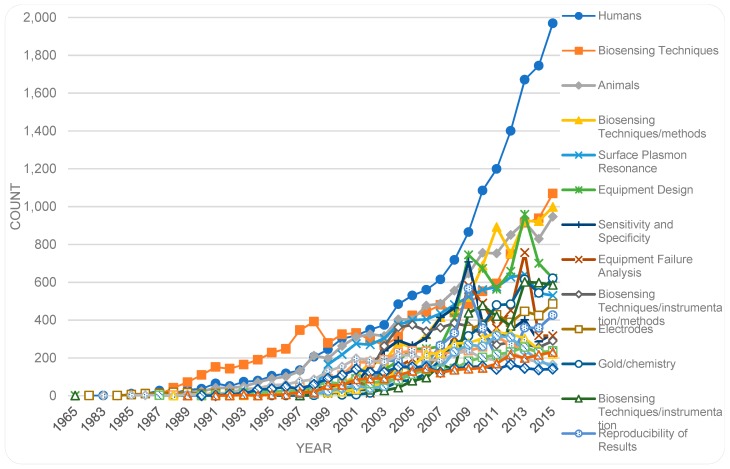
MeSH terms (top 20) by year.

We performed some pre-processing before analyzing the trends of the MeSH terms. For this, we removed the attached symbol of asterisk (*), which is used as an indicator of the centrality of the term in the given publications. If this was not done, the same term with and without the asterisk would have been recognized as two different terms. Following that, the numbers of occurrences of different terms were analyzed. The top 20 MeSH terms are displayed in descending order in Figure 17. A trend chart of the top terms by years is shown in Figure 18. The top terms include, e.g., *Humans*, *Biosensing Techniques*, *Animals*, *Biosensing Techniques/methods*, and *Surface Plasmon Resonance*. As indicated in Figure 18, bio-sensor-related publications, with MeSH term *Humans*, have increased steadily over the years. The number of publications with the term *Humans* exceeds by far, the number of publications with other terms in year 2015. Indeed, the number of publications related to the term *Humans* in year 2015 is by far more than the number of publications related to any other term in any year. The trends indicate a rather steady rise of publications associated to the MeSH terms *Humans, Biosensing Techniques* and *Animals*. Even publications associated with other terms such as *Biosensing Techniques/methods* have risen, although with some fluctuation. The fluctuations and sharp peaks become more noticeable in publications associated with terms such as *Equipment Design*, *Sensitivity and Specificity*, and *Equipment Failure Analysis.*

### 4.2. Keywords—Co-Occurrence Analyses Comparison

A study of keyword co-occurrences is a useful means of forming a sense of the core topics of discussions and network of conceptual relations. Both MeSH and Other terms were extracted and their occurrences were analyzed. Since MeSH Terms are controlled, the total number of unique *MeSH Terms* (12,456) in this data set was less than the total number of unique *Other Terms* (32,211). At the same time, many papers did not include *Other Terms* and hence as a result, the total number of occurrences of *MeSH Terms* (555,635) was by far more than the total number of occurrences of *Other Terms* (68,988).

For a sense of the overall use of keywords, co-occurrence analyses were conducted using the VOSviewer tool [38], which allows three units of analysis to be undertaken: (a) *MeSH keywords*, i.e., MeSH Terms, (b) *Author keywords*, i.e., Other Terms, and (c) *All keywords* (which is a combination of the former two keyword types).

To begin with, some pre-processing and data clean-up was performed using the thesaurus option provided by the tool. This pre-processing included, for example, a conversion of both the plural and singular forms of a *word* to “*word*(s)”, or the conversion of different versions of the same term, e.g., hyphenated, non-hyphenated and abbreviated forms, to one of the forms.

For the analyses, the minimum number of occurrences of keywords was set up to be “5”, the tool then calculated the total strength of co-occurrence links with other keywords. Based on this, the top 500 keywords were then selected to be included in the analyses and visualizations. Table 4 lists the top 20 most frequent MeSH terms, Other Terms, and All Keywords. In comparing these, there are three terms that appear in all three lists (i.e., *surface plasmon resonance*, *dna*, and *electrochemistry*). The information in Table 4 is an indication of the higher frequency of the MeSH terms in the data set. This can be seen, for example, by comparison of the number of occurrences listed, or the fact that the list of terms in All Keywords are to a large extent in the same order as the terms included in the MeSH Terms list.

Notably, the top ranked word in Other Terms, i.e., “biosensor(s)” takes the position 27 in the list of All Keywords and hence does not appear in the list of top 20 below. It should, however, be noted that some of the terms that are ranked lower than “biosensor(s)” in the list of Other Terms, for example “dna”, do appear in the list of top 20 terms in All Keywords. This is because they are also included in the MeSH Terms and therefore the summation of their occurrences both as MeSH and Other terms leads to their higher placement in the All Keywords list.

In the following three subsections, findings are presented, first on the collective of all keywords and then with a focus on each of the MeSH Terms and Other Terms.

### 4.3. Co-Occurrence Analyses of All Keywords

**Figure 19:**
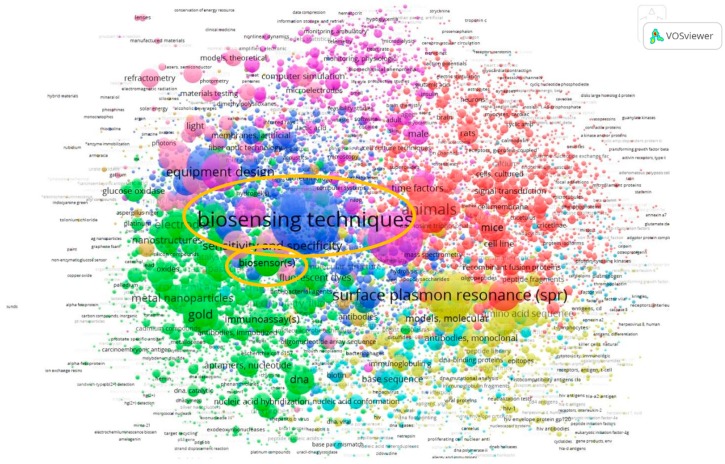
Co-occurrence analysis of All Keywords.

An analysis of All Keywords provides a sense of the level of use of different terms in the dataset. The keywords, with most frequent co-occurrences in MeSH and Other Terms are *biosensing techniques* and *biosensor(s)* respectively. Figure 19 depicts a map of co-occurrences of All Keywords. For this analysis the minimum number of occurrences of a term was set to 5, which meant that of 42,857 terms, in total 7145 terms met the threshold and hence are included in the figure. Similar to the indications in Table 4, this figure also highlights the dominance of the MeSH Terms in this material, where the top MeSH Term (biosensing techniques) is more dominant than the top Other Term (biosensor(s)).

### 4.4. Co-Occurrence Analysis of MeSH Terms

**Figure 20:**
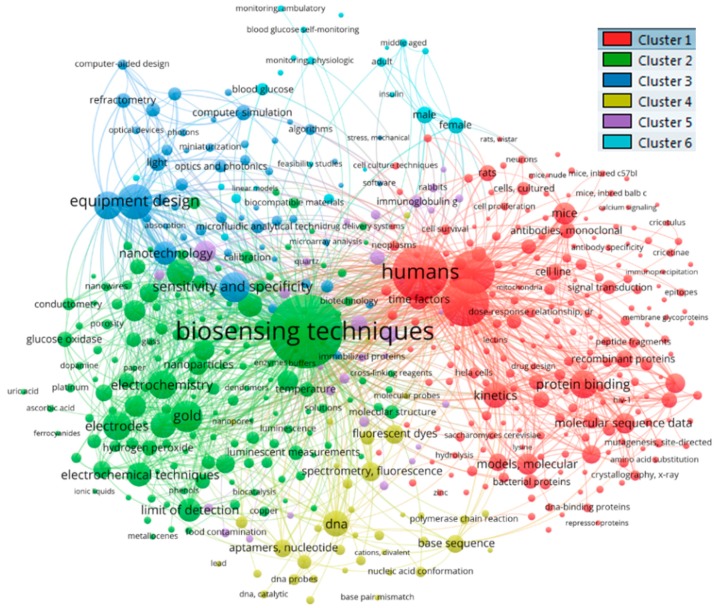
Co-occurrence analysis of MeSH Terms.

When conducting a co-occurrences analysis, by setting the minimum number of occurrences of a keyword to 5, of 12,546 terms, 5686 keywords meet the threshold and of these the top 500 with the greatest link strength are selected and depicted in Figure 20. There are six different clusters formed and Table 5 lists the top 20 keywords in each cluster.

By examining this network visualization more closely, some common denominators among the items in each cluster become evident. For example, the publications in cluster one often relate to subjects such as *humans*, *animals*, *mice*, and *rats*; the term *dna* is rather frequent in the items in cluster four, and demographic variables seem to be of interest in cluster six.

**Figure 21:**
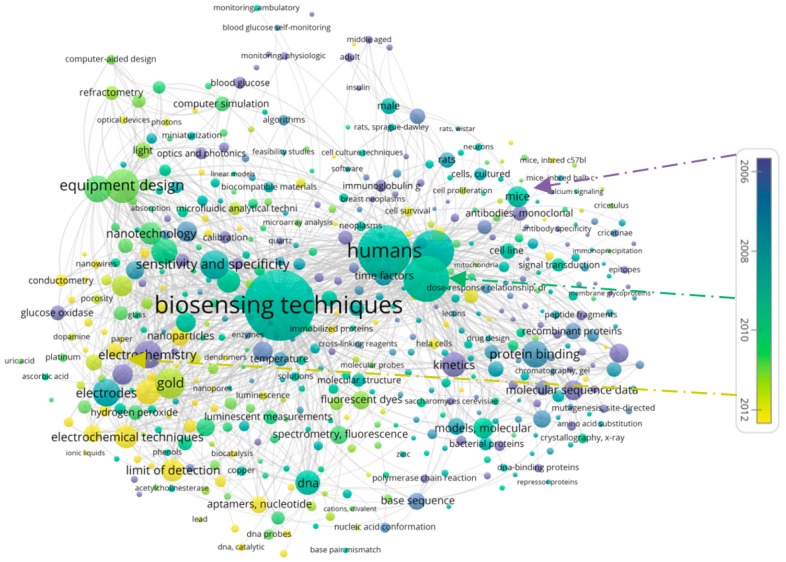
Co-occurrence analysis of MeSH Terms with a time overlay.

Typically, new terms emerge at different times and then referred to in varying degrees over the years. Some are used continually, some become dominant in periods, while others die away. It is difficult to form an understanding of the temporal trends in a field simply from a general overview of the co-occurrences of terms. To get a sense of the recent developments, Figure 21 is a variation of Figure 20, where the time dimension is overlaid with the use of colors. As shown, the more recent terms are marked with light green and yellow colors. 

The basis for this image is the average publication year. As shown, keywords colored in darker blue and purple denote keywords whose average year of publication goes back to the mid-2000s. Among the older keywords clearly visible in the diagram are for example *blood glucose*, *adult*, *middle aged*. A longer list of older keywords is shown in Table 6.

An examination of the terms in green (i.e., those with an average year of publication in middle of the color spectrum, e.g., *biosensing techniques*, *human*, *electrodes*) indicates that those terms have been continually used. Keywords marked in light green or yellow, however, have a more recent average year of appearance. Among these, as listed in Table 7, one can find terms *optical imaging*, *limit of detection*, and *electrochemical techniques*.

### 4.5. Co-occurrence Analysis of Other Terms

**Figure 22:**
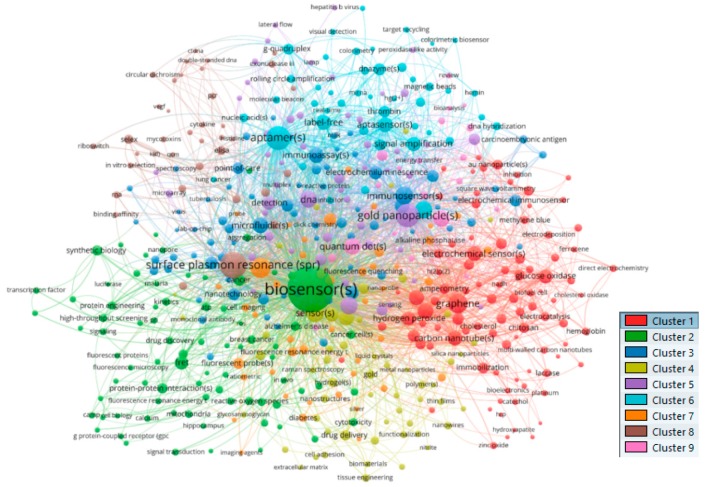
Co-occurrence analysis of Other Terms (or Author Keywords).

In contrast to the analysis of MeSH terms, in this section, we focus on the Other Terms (or Author Keywords). The same procedure is followed. By setting the threshold of minimum number of occurrences to 5; of 32,087 keywords, 1689 met the threshold and top 500 terms with greatest total link strength were selected. When clustering is done, the landscape looks somewhat different. Here, the terms “biosensor(s)” is the main keyword used by authors rather than “biosensing techniques”. There are now nine clusters formed as presented in Figure 22. With this in mind, the top 20 most frequent terms in each of the nine clusters are presented in Table 8.

**Figure 23:**
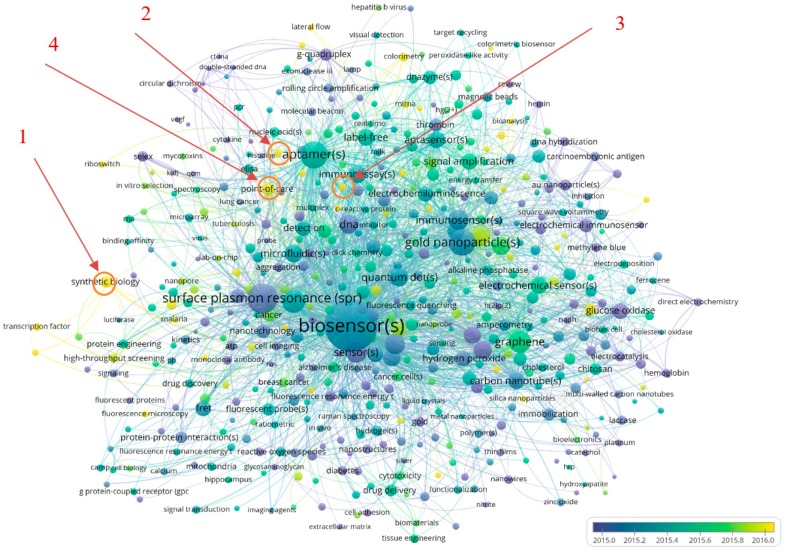
Co-occurrence analysis of Other Terms (or Author Keywords) with time overlay.

As mentioned earlier, Author keywords are only indexed since 2013 and, hence, they are not present in all items of the dataset affecting the average publication years. With that in mind, a temporal dimension is overlaid, by changing the color scheme (Figure 23). A list of the recent terms (with average year of publication being 2016) is provided in Table 9. In Figure 23, the top four terms that have emerged in more recent times (as presented in Table 9) have been marked with numbers including (1) synthetic biology, (2) smartphones, (3) fluorescent biosensor, and (4) electrochemical biosensor(s).

In addition to co-occurrence analyses, it would also be informative to identify the new keywords that emerge each year as assigned by the authors. Table 10 presents a list of newer author keywords that have occurred 40 or more times.

## 5. Discussion and Conclusions

This study has provided an overview of the patterns of scholarly publications on the topic of biosensor(s). The study indicates a steady growth in related publications, with a lower rate of growth in recent years which needs to be investigated further at a later date to allow for potential time lapse in registration. When analyzing total numbers per country, contributions by authors from the US, China, followed by Japan, Germany, UK, and South Korea are dominant in the field. A breakdown of the analysis by countries over time shows a slowdown of publications on the topic in the US, while publications by China-based authors have continued to evolve with upward trends, holding the top position since 2013. The decline in the number of publications by US-based authors in recent years raises questions requiring further investigation. Have new concepts or subdomains evolved that are being written about instead? The recent dominance of publications by Chinese authors is in line with studies (e.g., [41]) that have reported an increased scientific productivity in China, positioning it as a top contributor to science globally. Occupation of the top position since 2013 may be indicative of early interest in this topic by China, and it is worth further investigation and comparison with the temporal order of Chinese dominance in other fields (e.g., sensors in general). Another finding is that although the top three publication places are the US, the UK, and the Netherlands, the number of publications, especially by the UK- and Netherlands-based authors, do not follow this pattern. In other words, while these countries host a large portion of publishing outlets, the researchers based there produce a lesser portion of the actual publications. This trend is reversed in some other countries, such as South Korea, where the country ranks relatively low as a publishing outlet, but high if the number of publications by the authors located in that country is considered. A further finding is that while publications by US-based authors are dominant in terms of total numbers of publications per country, publications by EU-based authors as a collective are significantly larger in numbers than either of those published by the US-based or China-based authors. Furthermore, if we look at the level of contributions per country per capita, then Singapore takes the first position followed by Sweden, Switzerland and Denmark, while the US and China are ranked lower, i.e., in positions 8 and 19 respectively.

The data in this study indicates that the majority of publications on the topic are of type ‘journal articles’ and the top journals in which these articles are published are *Biosensors & bioelectronics, Analytical chemistry, Optics express* and *Sensors.* In analyses of publication types, the data even provided an initial indication of different support forms, (e.g., governmental, non-governmental, etc.). A question that arises for future studies is an examination of the support provided by public funding in each country in comparison with the level of publications by scholars in those countries.

Top authors in the field have also been examined and presented. A finding was that some of the authors with most publications appeared less as the first author, while some of the authors with high but slightly fewer number of publications had a significant presence both as first and last authors. The position in author lists varies from discipline to discipline. With large number of authors in some papers (the highest number being over 150 authors in one of the papers), it would be interesting to examine the role and the level of contributions of the authors to the contents of the publications in general and in connection to their position in author lists in particular.

In examining the number of publications related to authors, journals, countries and regions, a distribution with a long tail has been the norm, where the top few have been related to a large number of publications. Moreover, a large number have collectively been associated with a smaller number of publications, as indicated by the core distribution laws of bibliometrics.

The study has also examined in some detail the keywords associated with the publications. Due to the choice of database, MeSH terms have a larger presence in this dataset. In a co-occurrence analysis, the top five MeSH terms were identified as *biosensing techniques*, *humans*, *surface plasmon resonance*, *animals*, and *equipment design*. In a similar analysis of Other Terms, the top five keywords were identified to be *biosensor(s)*, *surface plasmon resonance*, *aptamer(s)*, *gold nanoparticle(s)*, and *fluorescence*. Moreover, we identified various clusters or grouping of different topics within the publications and by overlaying a time dimension on the findings, a temporal sense of emerging topics over the years has been presented.

These findings are indicative of developments in the field. We hope that these findings may be of use to scholars in the biosensors field. Due to the interest in MeSH Terms and other considerations, the main dataset used for this study was extracted from the PubMed database which includes publications on biosensor with some bias privileging biomedicine and biomedical applied biosensors. Furthermore, the dataset did not include citation information. It would be of interest to conduct a similar study based on the data extracted from other sources, including both indexed citation databases and web-based unstructured data to provide further insights as to the impact of the publications in terms of reach and citations. Moreover, further studies could provide access to more recent developments based on web-based discussions, other discourse, and conference presentations that have not yet been formally published.

## Figures and Tables

**Table 1:** Overview of searches conducted in WoS on 8 May 2019.

Search Term	Results
Topic:(biosensor*) OR TOPIC: (bio-sensor*)	85,174
TOPIC: (sensor*)	1,039,670
PUBLICATION NAME: (sensors)	18,997
PUBLICATION NAME: (biosensors & bioelectronics)	11,794

**Table 2:** List of the dominant countries with 500 or more publications based on the first author’s affiliation (PubMed dataset, all years).

First Author Affiliation Country	Count of Publications	First Author Affiliation Country	Count of Publications
United States	13,019	Taiwan, Province of China	1134
China	10,420	Sweden	1053
Japan	3023	Australia	882
Germany	2936	Switzerland	756
United Kingdom	2502	Iran, Islamic Republic of	753
Korea, Republic of	2279	Singapore	699
France	1860	N/A*	650
Italy	1624	Netherlands	626
Spain	1524	Israel	503
India	1490	Denmark	502
Canada	1436	Total	47,390

* In the PubMed dataset, 1989 items did not include affiliation or country information. We managed to find the missing information for 1339 of those items by cross-referencing the available information (e.g., title, author, doi) with data in the WoS database and by manually accessing some of the publications. Despite our efforts we were not able to find country information for 650 items (e.g., [42]); these are marked with N/A (not available) in Table 2.

**Table 3:** List of top ten authors in the first five co-authorship clusters.

Cluster 1	Cluster 2	Cluster 3	Cluster 4	Cluster 5
zhang, lei	wang, jing	wang, joseph	zhang, hua	tan, weihong
wang, lei	wang, li	xiao, yi	wang, yi	li, jinghong
wang, hui	li, feng	plaxco, kevin w	zhang, juan	wang, kemin
liu, chang	li, wei	carrara, sandro	dong, shaojun	yu, ru-qin
zhu, jun-jie	wang, ying	gorton, lo	wang, jian	huang, jin
xu, hui	yang, fan	kerman, kagan	zhou, ming	shen, guo-li
wang, feng	liu, tao	ozsoz, mehmet	chen, peng	wang, hua
li, chen-zhong	wang, qian	mulchandani, ashok	niu, li	yu, ruqin
li, yang	li, genxi	tkac, jan	wang, erkang	zhang, xiao-bing
liu, guodong	li, hui	antiochia, riccarda	zhang, peng	shen, guoli

**Table 4:** Top 20 most frequent Terms in the dataset.

Rank	MeSH Terms	Freq.	Other Terms	Freq.	All Keywords	Freq. ^1^
1	biosensing techniques	32,646	biosensor(s)	2577	biosensing techniques	32,652
2	humans	18,333	surface plasmon resonance (spr)	667	humans	18,333
3	surface plasmon resonance	13,463	aptamer(s)	501	surface plasmon resonance (spr)	13,580
4	animals	11,540	gold nanoparticle(s)	476	animals	11,541
5	equipment design	7385	fluorescence	339	equipment design	7385
6	gold	5982	graphene	300	gold	5996
7	sensitivity and specificity	5539	sensor(s)	265	sensitivity and specificity	5539
8	electrodes	4937	immunosensor	247	electrochemistry	5051
9	electrochemistry	4907	nanoparticle(s)	234	electrodes	4941
10	equipment failure analysis	4653	electrochemical biosensor(s)	230	protein binding	4654
11	protein binding	4649	dna	220	equipment failure analysis	4653
12	reproducibility of results	4370	quantum dot(s)	200	reproducibility of results	4370
13	metal nanoparticles	4034	graphene oxide	194	metal nanoparticles	4040
14	kinetics	3913	microfluidic(s)	185	dna	3972
15	dna	3874	glucose	173	kinetics	3931
16	surface properties	3574	electrochemistry	166	surface properties	3574
17	electrochemical techniques	3494	biosensing	165	electrochemical techniques	3495
18	nanotechnology	3410	immunoassay(s)	162	nanotechnology	3468
19	limit of detection	3328	label-free	154	limit of detection	3341
20	mice	3023	aptasensor(s)	153	mice	3023

^1^ It should be observed that since some of the terms (e.g., dna) appear in the same paper both as MeSH Terms and Other Terms, the frequency of their appearance in the *All Keywords* column would not be the sum of the frequency of their appearance in the other two terms. Some of the terms have been color-coded to facilitate comparison of their position in the list of MeSH terms and All Keywords.

**Table 5:** Top 20 terms in each of the clusters that are formed with co-occurrence analysis of MeSH Terms.

	Cluster 1	Cluster 2	Cluster 3	Cluster 4	Cluster 5	Cluster 6
1	humans	biosensing techniques	equipment design	dna	immunoassay	male
2	surface plasmon resonance	gold	sensitivity and specificity	spectrometry, fluorescence	cattle	female
3	animals	electrodes	equipment failure analysis	fluorescent dyes	proteins	blood glucose
4	protein binding	electrochemistry	nanotechnology	base sequence	antibodies	biomarkers
5	kinetics	reproducibility of results	light	aptamers, nucleotide	immunoglobuling	biomarkers, tumor
6	mice	metal nanoparticles	computer simulation	nucleic acid hybridization	serum albumin, bovine	adult
7	molecular sequence data	surface properties	scattering, radiation	fluorescence	antibodies, immobilized	middle aged
8	models, molecular	electrochemical techniques	refractometry	colorimetry	biotin	monitoring, physiologic
9	amino acid sequence	limit of detection	materials testing	dna probes	streptavidin	blood chemical analysis
10	time factors	enzymes, immobilized	models, chemical	nucleic acid conformation	rabbits	prostate-specific antigen
11	binding sites	hydrogen-ion concentration	microfluidic analytical techniques	dna, single-stranded	immobilized proteins	point-of-care systems
12	recombinant proteins	nanostructures	crystallization	oligonucleotides	protein array analysis	aged
13	protein structure, tertiary	nanoparticles	optics and photonics	thrombin	antigen-antibody reactions	blood glucose self-monitoring
14	escherichia coli	oxidation-reduction	models, theoretical	oligonucleotide array sequence analysis	food analysis	feasibility studies
15	cell line	polymers	silicon	polymerase chain reaction	bacteria	insulin
16	protein conformation	glucose	transducers	dna, bacterial	food contamination	linear models
17	ligands	graphite	computer-aided design	g-quadruplexes	biotinylation	prostheses and implants
18	rats	nanotubes, carbon	electric impedance	dna, catalytic	anti-bacterial agents	monitoring, ambulatory
19	antibodies, monoclonal	hydrogen peroxide	metals	nucleic acid amplification techniques	quartz	prostatic neoplasms
20	cell line, tumor	glucose oxidase	algorithms	mercury	milk	diabetes mellitus

**Table 6:** Older MeSH Terms among those depicted in Figure 21.

Term	Occurrence	Avg. Publ. Year
antigen-antibody complex	210	1976
diabetes mellitus, type 1	168	1983
prostheses and implants	237	1989
biotechnology	596	1991
immunoglobulin variable region	162	1991
cross reactions	165	1993
rna, messenger	273	1994
membrane potentials	153	1994
organometallic compounds	326	1996
urea	212	1996
ferrous compounds	348	1997
indicators and reagents	340	1997
blood glucose	965	1999
antigen-antibody reactions	441	1999
insulin	254	1999
oxygen	878	2000
in vitro techniques	475	2000
microchemistry	334	2000
hiv-1	285	2000
serum albumin	261	2000
metallocenes	232	2000

**Table 7:** Newer MeSH Terms among those depicted in Figure 21.

Term	Occurrences	Avg. Publ. Year
optical imaging	216	2015
limit of detection	3328	2014
hek293 cells	517	2014
micrornas	326	2014
magnetite nanoparticles	302	2014
paper	216	2014
mcf-7 cells	170	2014
electrochemical techniques	3494	2013
graphite	1938	2013
aptamers, nucleotide	1852	2013
oxides	863	2013
nanocomposite(s)	693	2013
antibodies, immobilized	692	2013
dielectric spectroscopy	614	2013
molecular imprinting	424	2013
nucleic acid amplification techniques	414	2013
g-quadruplexes	407	2013
lab-on-a-chip devices	399	2013
quartz crystal microbalance techniques	312	2013
molecular imaging	301	2013
high-throughput screening assays	271	2013
nanopores	235	2013
drug discovery	201	2013
photoelectron spectroscopy	198	2013
molecular dynamics simulation	197	2013
enzyme assays	195	2013
polymerization	193	2013
young adult	152	2013

**Table 8:** The top 20 most frequent *Other Terms* in each of the clusters depicted in Figure 22. Grey shades are added to rows to improve readability.

Cluster 1	Cluster 2	Cluster 3	Cluster 4	Cluster 5	Cluster 6	Cluster 7	Cluster 8	Cluster 9
graphene	biosensor(s)	immunosensor(s)	sensor(s)	gold nanoparticle(s)	aptamer(s)	fluorescence	surface plasmon resonance (spr)	quantum dot(s)
glucose	fret	microfluidic(s)	biosensing	nanoparticle(s)	electrochemical biosensor(s)	localized surface plasmon resonance (lspr)	selex	graphene oxide
electrochemistry	cancer	immunoassay(s)	self-assembly	dna	label-free	fluorescent probe(s)	quartz crystal microbalance	electrochemical detection
carbon nanotube(s)	protein-protein interaction(s)	electrochemical impedance spectroscopy (eis)	sers	detection	aptasensor(s)	gold nanorod(s)	elisa	sensitivity
electrochemical sensor(s)	synthetic biology	nanomaterial(s)	drug delivery	dna biosensor	signal amplification	fluorescence resonance energy transfer	protein	dna methylation
glucose oxidase	imaging	electrochemiluminescence	hydrogel(s)	optical biosensor(s)	dnazyme(s)	glutathione	luminescence	point-of-care testing
hydrogen peroxide	alzheimer’s disease	antibody	plasmonics	silver nanoparticles	thrombin	nanosensor(s)	molecular recognition	photoelectrochemistry
molecularly imprinted polymer(s)	breast cancer	electrochemical	field-effect transistor(s)	label-free detection	microrna	fluorescence quenching	bovine serum albumin	photoelectrochemical
dopamine	cancer cell(s)	biomarker(s)	gold	impedance	g-quadruplex	alkaline phosphatase	oligonucleotide(s)	selectivity
screen-printed electrode(s)	photonic crystal(s)	electrochemical immunosensor	atomic force microscopy	bacteria	dna detection	bioimaging	small molecule(s)	electrochemical immunoassay
immobilization	reactive oxygen species	nanotechnology	nanostructures	escherichia coli	hybridization chain reaction	diabetes	exonuclease iii	refractive index
amperometry	protein engineering	point-of-care	biocompatibility	colorimetric detection	colorimetric	glucose sensor	rna	energy transfer
cyclic voltammetry	apoptosis	peptide(s)	heparin	food safety	nucleic acid(s)	carbon dots	pcr	review
nanocomposite(s)	atp	magnetic nanoparticle(s)	surface functionalization	pesticide(s)	magnetic beads	cell imaging	circular dichroism	metal ions
chitosan	cytotoxicity	diagnosis	electrospinning	e. coli	nanobiosensor	graphene quantum dots	in vitro selection	tumor marker
self-assembled monolayer(s)	glutamate	chemiluminescence	nanowires	microbial fuel cell	ochratoxin a	cysteine	mycotoxins	bioanalysis
reduced graphene oxide	kinetics	voltammetry	polymer(s)	antibodies	dna hybridization	mercury	riboswitch	liquid crystal
differential pulse voltammetry	high-throughput screening	diagnostics	tissue engineering	microarray	electrochemical aptasensor	photoluminescence	binding affinity	screen-printed carbon electrode
glucose biosensor	oxidative stress	cancer biomarker(s)	functionalization	toxicity	rolling circle amplification	flow cytometry	concanavalin a	signal enhancement
au nanoparticle(s)	adenosine	impedance spectroscopy	surface modification	pathogen detection	protein detection	fluorescent sensor	cytokine	enzyme catalysis

**Table 9:** The top 30 Terms with more recent ‘average publication years’ included in Figure 14.

Rank	Keyword	Occurrence	Rank	Keyword	Occurrence
1	synthetic biology	62	16	lateral flow	15
2	smartphone	30	17	mycobacterium tuberculosis	15
3	fluorescent biosensor	27	18	food analysis	14
4	point-of-care testing	27	19	malaria	14
5	mirna	23	20	pencil graphite electrode	14
6	metal-organic frameworks	21	21	silica nanoparticles	14
7	transcription factor	20	22	tumor marker	14
8	drug screening	18	23	biomolecules	13
9	luminol	18	24	quantification	13
10	environmental monitoring	17	25	ultrasensitive	13
11	optical	17	26	bioanalysis	12
12	living cells	16	27	molybdenum disulfide	12
13	metabolic engineering	16	28	screen-printed carbon electrode	12
14	riboswitch	16	29	terminal deoxynucleotidyl transferase	9
15	evanescent wave	15	30	indium tin oxide	7

**Table 10:** Recent author keywords based on average publication years and the number of occurrences.

**Average publication year: 2015**	
Nucleic Acid Amplification Techniques	42
Molecular Imprinting/methods	40
Gene Expression	40
**Average publication year: 2014**	
Ions	41
**Average publication year: 2013**	
Electrochemical Techniques/instrumentation	68
Fiber Optic Technology/instrumentation	56
Energy Transfer	50
Thrombin/analysis	43
Systems Integration	41
DNA, Single-Stranded/chemistry	41
**Average publication year: 2012**	
Hydrophobic and Hydrophilic Interactions	43
Absorption	41
Telecommunications/instrumentation	40
**Average publication year: 2011**	
Biosensing Techniques/methods/statistics & numerical data	69
Dielectric Spectroscopy	57
Chitosan/chemistry	46
X-Ray Diffraction	43
Nanocomposites/chemistry	42
